# Multimodal ECG heartbeat classification method based on a convolutional neural network embedded with FCA

**DOI:** 10.1038/s41598-024-59311-0

**Published:** 2024-04-16

**Authors:** Feiyan Zhou, Duanshu Fang

**Affiliations:** 1 Key Lab of Education Blockchain and Intelligent Technology, Ministry of Education, Guangxi Normal University, Guilin, 541004 China; 2 Guangxi Key Lab of Multi-Source Information Mining and Security, Guangxi Normal University, Guilin, 541004 China

**Keywords:** ECG, Multi-modal fusion, Classification, Convolutional neural network, Frequency-channel attention, Computational biology and bioinformatics, Cardiology, Diseases

## Abstract

Arrhythmias are irregular heartbeat rhythms caused by various conditions. Automated ECG signal classification aids in diagnosing and predicting arrhythmias. Current studies mostly focus on 1D ECG signals, overlooking the fusion of multiple ECG modalities for enhanced analysis. We converted ECG signals into modal images using RP, GAF, and MTF, inputting them into our classification model. To optimize detail retention, we introduced a CNN-based model with FCA for multimodal ECG tasks. Achieving 99.6% accuracy on the MIT-BIH arrhythmia database for five arrhythmias, our method outperforms prior models. Experimental results confirm its reliability for ECG classification tasks.

## Introduction

Cardiovascular diseases (CVDs) are a significant global health concern, responsible for an estimated 17.9 million deaths annually^1^. The high prevalence of CVDs leads to substantial medical expenses^2^. Electrocardiogram (ECG) analysis plays a pivotal role as a non-invasive diagnostic tool for cardiovascular ailments^3^. An ECG signal typically showcases four fundamental waveforms: the P wave, QRS complex, T wave, and U wave^4^. Nonetheless, due to the intricate and dynamic nature of ECG signals, classifying ECG heartbeats poses a challenge for researchers^5^. As such, the development of intelligent diagnostic systems is paramount in advancing cardiology^6^.

Early approaches to heartbeat classification using ECG signals relied on signal processing^7^and statistical techniques^8^for feature extraction. The strength of these conventional methods lies in their ability to segregate feature extraction from pattern classification. In recent years, deep learning has exhibited remarkable performance and brought about significant innovations across various domains, including computer vision^9^, natural language processing^10^, strategic games^11^, and medical fields^12^. Notably, research efforts have focused on leveraging deep learning techniques for automatic feature learning and ECG beat classification^13^, utilizing models such as Deep Neural Networks (DNN)^14^, Convolutional Neural Networks (CNN)^15^, Recurrent Neural Networks (RNN)^16^, and Generative Adversarial Networks (GAN)^17^ to analyze arrhythmia and ECG signals. Yuanlu Li et al.^18^designed an improved residual network for arrhythmia classification and proposed an overlapping segmentation method to overcome the problem of inter-class data imbalance. Oh et al.^19^ proposed an automatic system that uses a combination of CNN and Long Short-term Memory (LSTM) for arrhythmia detection to handle ECG signals of different lengths. Yang, F et al.^20^ proposed a convolutional block called PDblock, which consists of pointwise convolutional layers and deep convolutional layers, and used a loss function to improve arrhythmia classification results, achieving good results on the MIT-BIH arrhythmia (MIT-BIH-AR) database. The aforementioned methods primarily focus on treating ECG data as a 1D time series. Nevertheless, some researchers have identified limitations in these conventional approaches. Few previous methods have explored how to integrate multiple modules to inherit the advantages of time series. Chen et al.^21^ introduced a cross-modal data processing method, which not only enhances model performance but also improves model robustness. In a similar vein, Han et al.^22^ transformed ECG signals into GAF images, integrating them with the original ECG signals as multimodal inputs which enables the model to learn complementary information between different modalities. Furthermore, Yang et al.^23^ considered different leads as distinct views, effectively leveraging the diversity of the 12 lead features and achieving commendable outcomes in multi-label tasks.

The above research results strongly indicate that the integration of multiple modalities in ECG analysis helps overcome the limitations of individual modalities, enhancing the accuracy of analysis and classification tasks^24^. This study employs three distinct data conversion methods to transform 1D ECG signals into three 2D datasets, extracting valuable information and high-dimensional features suitable for nonlinear classifiers. A novel classification method for arrhythmia, combining deep residual CNN and frequency channel attention (FCA)^25^, is introduced to address the issue of insufficient channel attention information, thereby enhancing the ECG classification model’s performance. Various techniques like Noise Augmentation, Geometric Transformation, and other data augmentation methods are extensively employed in the realm of deep learning. Among these, SMOTE (Synthetic Minority Over-sampling Technique) stands out as a method that creates a new sample in each direction by randomly selecting the k nearest neighbors from the minority class. The fundamental concept underlying this approach is not to alter the data itself but to create fresh data derived from the original dataset. While the Borderline-SMOTE^26^ technique utilized in this study is conceptually akin to SMOTE, it only over-sampling or reinforcing the minority instances situated at the borderline. The effectiveness of the proposed method is validated using the MIT-BIH-AR database, exhibiting significant enhancements in experimental results.

The subsequent sections of this paper are organized as follows: “Material and method” delineates the proposed model structure, “Experiments” presents experimental details, “Result and discussion” discusses the results, and “Conclusion” concludes the study.

## Material and method

### Material

#### ECG database

This study utilizes the MIT-BIH-AR database^3^. As shown in Table 1, The arrhythmias are categorized into five types based on the Association for the Advancement of Medical Instrumentation (AAMI)^27^ standard, which includs normal (N), supraventricular ectopic beat (SVEB), ventricular ectopic beat (VEB), fusion beat (F), and unknown beat (Q) categories^28^. The MIT-BIH-AR database comprises 48 half-hour-long ECG recordings sampled at a rate of 360 Hz from 47 different subjects. Over 110,000 annotations were independently reviewed and annotated by two or more cardiac experts. Furthermore, each recording includes two ECG derivations, with only lead II utilized in this study.

**Table 1:** AAMI recommended classes for heartbeats.

Class	Symbol	Members
Normal	N	Normal beat Left bundle branch block beat Right bundle branch block beat Atrial escape beat Nodal(junctional)escape beat
Supraventricular Ectopic Beat	SVEB	Atrial premature beat Aberrated atrial premature beat Nodal (junctional) premature beat Supraventricular premature or ectopic beat (atrial or nodal)
Ventricular ectopic beat	VEB	Premature ventricular contraction Ventricular escape beat
Fusion beat	F	Fusion of ventricular and normal beat
Unknown beat	Q	/Paced beat Fusion of paced and normal beat Unclassifiable beat

#### ECG preprocessing

**Figure 1:**
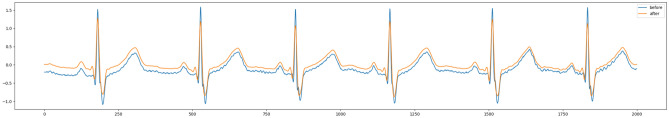
The ECG signals from the MIT-BIH-AR database before and after filtering with the bandpass filter.

Preprocessing the raw obtained ECG signals is necessary, as they are often contaminated by different types of noise, such as baseline drift, power line interference, and patient electrode motion artifacts^29^. In order to enhance the signal-to-noise ratio (SNR) and streamline R-peak detection and heartbeat classification, this study preprocesses the raw ECG signals by employing a bandpass filter with a frequency range of 0.5 to 50 Hz to reduce noise levels. The waveforms of both the original and filtered ECG signals can be observed in Fig. 1.

We utilize the R-peak annotations from the MIT-BIH-AR database for heartbeat classification. Each R-peak annotation is associated with 324 samples, including 144 samples before and 180 samples after the peak, capturing the complete heartbeat. Subsequently, the filtered ECG signals undergo resampling to 224 Hz before proceeding to the subsequent stage for additional processing.

### ECG signal to image transformation

The input for the proposed model involves converting the ECG signal heartbeats into RP, GAF, and MTF images.

#### Image formation by a recurrence plot (RP)

Recurrence networks, derived from nonlinear time series, can extract hidden features from complex dynamic systems. Among the main methods for analyzing nonlinear time series networks, the recursive network method is an important tool for studying such complex systems^30^. Let $q(t)\in R^d$ be a multivariate time series; then, a recurrence network can be defined as follows:

1 $$\begin{aligned} RP=\theta (\varepsilon -||q(i)-q(j)||) \end{aligned}$$

**Figure 2:**
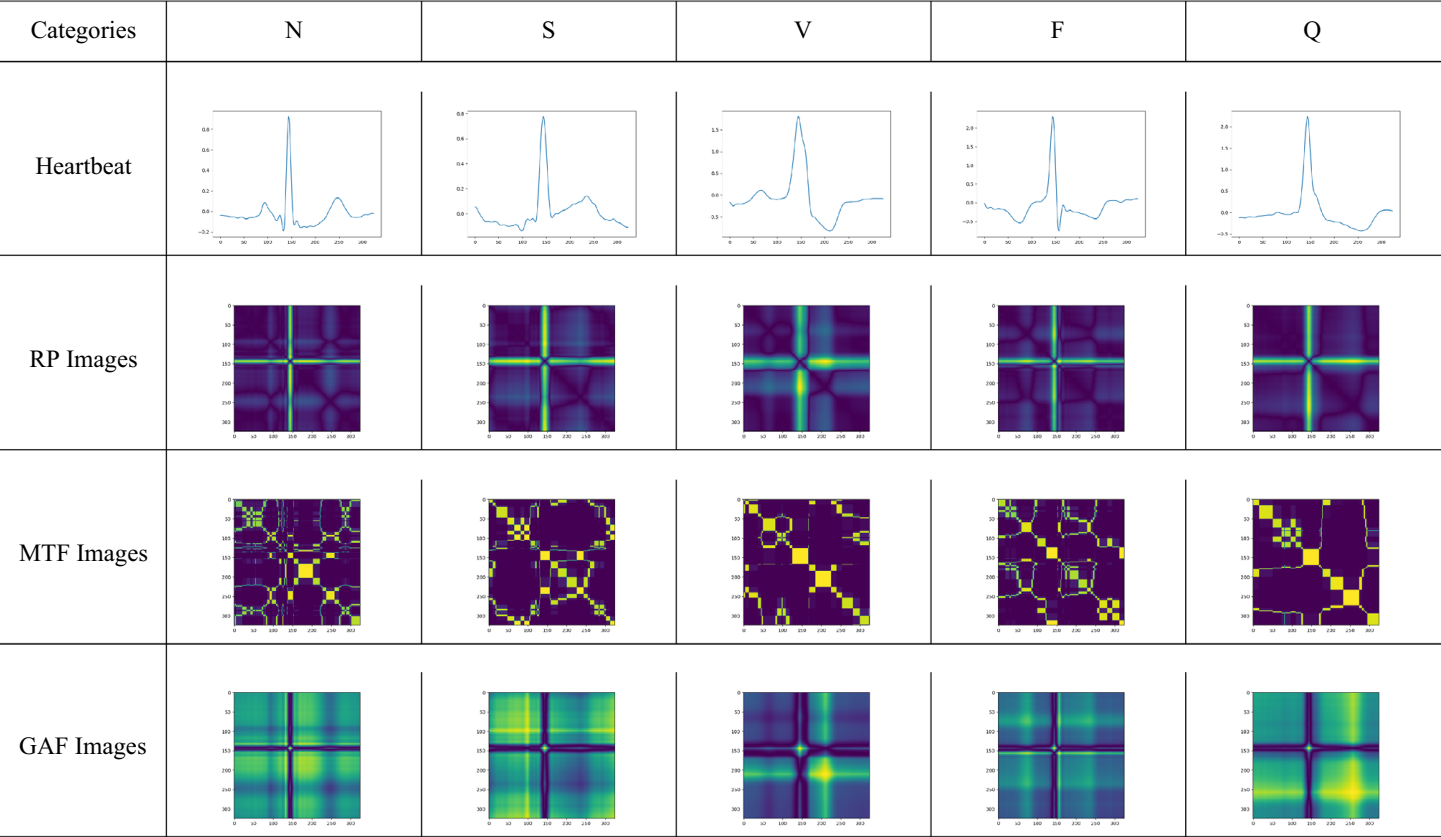
RP, MTF and GAF images of MIT-BIH-AR dataset according to the five different heartbeats.

In Eq. (1), $\varepsilon$ is the threshold and $\theta$ is referred to as the weight function. The heartbeat images transformed by RP are shown in Fig. 2.

#### Image formation by a gramian angular field

A gramian angular field (GAF) transforms 1D time series into 2D images through three steps, scaling, coordinate axis transformation, and trigonometric functions, thereby applying computer vision techniques to time series analysis^31^. Assuming a time series$X=\left\{ x_{1},x_{2},...x_{i},...x_{N}\right\}$, first, X is normalized so that all its values are between $[-1,1]$ or [0, 1], which can be respectively expressed as:

2 $$\begin{aligned} x_{-1}^{~i}= & {} \frac{(x_{i}-max(X))+(x_{i}-min(X))}{max(X)-min(X)}, \end{aligned}$$

3 $$\begin{aligned} x_{0}^{~i}= & {} \frac{x_{i}-min(X)}{max(X)-min(X)}. \end{aligned}$$

Afterward, the scaled sequence data is transformed into a polar coordinate system according to Eq. (4), where the values are treated as the cosine values of the angles, and the timestamps are treated as the radii.

4 $$\begin{aligned} {\left\{ \begin{array}{ll}{rcl} \phi =arccos(x_{i}^{~}),0\le x_{i}^{~},x_{i}^{~}\in X^{~} \\ r=\frac{t_{i}}{N},t_{i}\in {N} \end{array}\right. } \end{aligned}$$

A Gramian angular summation field (GASF) and a Gramian angular difference field (GADF) are defined as follows:

5 $$\begin{aligned} GAD{F}_{i,j}= & {} \cos \left( {\phi }_{i}-{\phi }_{j}\right) ,\quad {\forall }_{i,j}\in \left\{ 1,2,...,n\right\} \end{aligned}$$

6 $$\begin{aligned} GAS{F}_{i,j}= & {} \cos \left( {\phi }_{i}+ {\phi }_{j}\right) ,\quad {\forall }_{i,j}\in \left\{ 1,2,...,n\right\} \end{aligned}$$

#### Image formation by a Markov transition field

Markov chains can be used to model state-to-state transitions in a system^32^. The Markov Transition Field is an improvement based on the first-order Markov chain, which overcomes the problem of insensitivity to sequential temporal correlations in the Markov transition matrix. Assume a time series $X=\left\{ x_{1},x_{2},...x_{i},...x_{N}\right\}$the values can be quantized in *Q* bins , and each $x_{i}$ can be allocated to a related $q_{j}(j\in [1,{\mathbb {Q}}])$. By calculating the transitions among bins in the way of a first-order Markov chain along each time step, a matrix *W* of *Q* x *Q* size is obtained. $w_{i,j}$ is the probability that an element in $q_{j}$ is followed by an element in $q_{i}$. After normalization by $\sum _{j=1}^{Q}w_{ij}=1$, *W* is considered to be the Markov transition matrix. Since the matrix is not sensitive to the distribution of *X* and time steps $t_{i}$, in order to reduce the loss of information, the $M_{ij}$ in the Markov transition field (MTF) is defined as follows:

7 $$\begin{aligned} M_{ij}=\begin{bmatrix} w_{ij}|x_{1}\in q_{i},x_{1}\in q_{j}&{} \cdots &{} w_{ij}|x_{1}\in q_{i},x_{n}\in q_{j}\\ w_{ij}|x_{2}\in q_{i},x_{1}\in q_{j}&{} \cdots &{} w_{ij}|x_{2}\in q_{i},x_{n}\in q_{j}\\ \vdots &{} \ddots &{} \vdots \\ w_{ij}|x_{n}\in q_{i},x_{1}\in q_{j}&{} \cdots &{} w_{ij}|x_{n}\in q_{i},x_{n}\in q_{j}\\ \end{bmatrix} \end{aligned}$$

The Markov transition field (MTF) then can be defined as follows:

8 $$\begin{aligned} M=\begin{bmatrix} M_{11}&{} \cdots &{} M_{1n}\\ M_{21}&{} \cdots &{} M_{2n}\\ \vdots &{} \ddots &{} \vdots \\ M_{n1}&{} \cdots &{} M_{nn}\\ \end{bmatrix} \end{aligned}$$

The heartbeat images transformed by MTF are shown in Fig. 2.

### FCA Block

**Figure 3:**
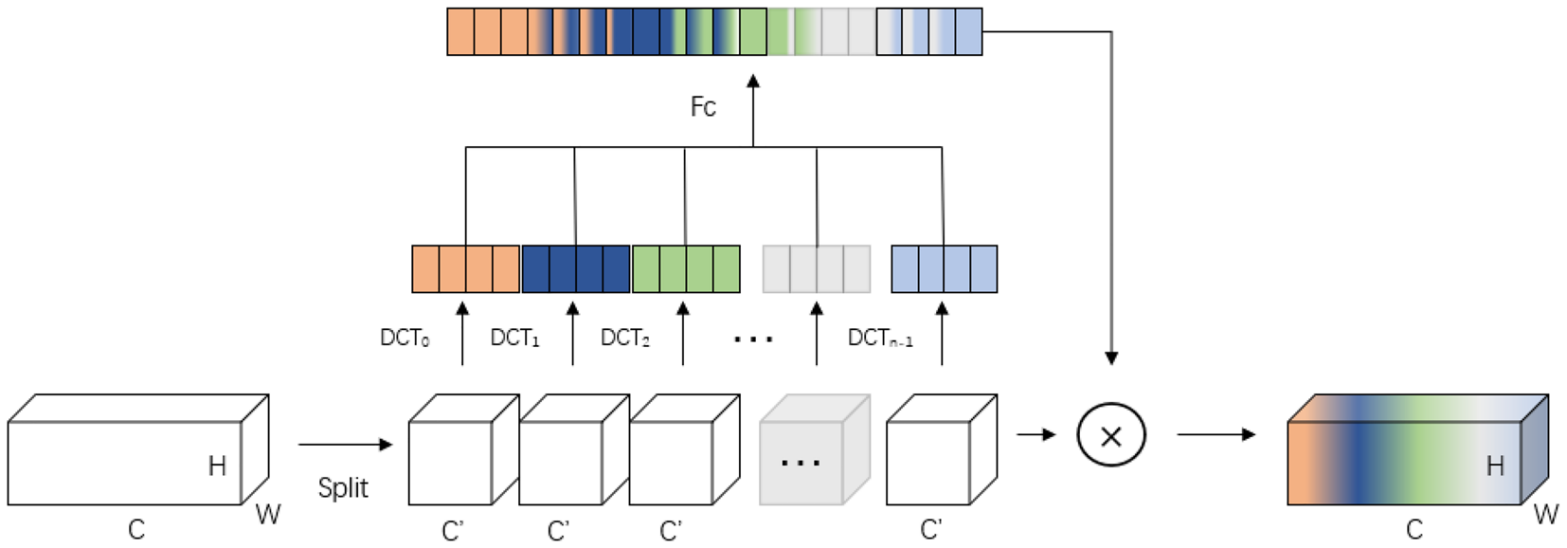
Schematic diagram of FCA structure.

FCA serves as an attention mechanism utilized in image processing to dynamically modify the weight of the input feature map, enhancing the extraction of information across various frequency channels. The configuration of the FCA mechanism is illustrated in Fig. 3. FCA is an extension of the channel attention mechanism with multiple spectral channels and proposes a “two-step” method for selecting different frequency domain components and different frequency combinations, which is used to extract different spectral features in different channels to achieve the purpose of obtaining richer information, which is consistent with the idea of converting one-dimensional ECG signals into spectrograms adopted in this study. Meanwhile, to solve the problem of deep learning networks focusing on low-frequency information, 2D discrete cosine transform (DCT) is used to compress channels in the attention mechanism, which focuses on low frequency without discarding other frequency components. The ECG signal is a combination of low and high frequency signals and the spectrograms derived from this transformation will be useful. The Ablation Study section of the article compares it with several other attention mechanisms, which strongly supports the above argument. In the FCA mechanism, the input feature map undergoes decomposition into various frequency channels. Subsequently, each channel is inputted into a fully connected layer to produce a scalar weight represented by equation(9). These weights are then utilized to modulate the respective frequency channels and produce weighted feature maps.

The weighted feature maps are consolidated and transmitted to the subsequent network layer for additional processing, denoted by Eq. (10). The FCA mechanism offers the benefit of adaptively modifying the weights across diverse frequency channels to enhance model performance. Furthermore, it provides excellent interpretability by explicitly illustrating the model’s focus on various frequency channels.

9 $$\begin{aligned}{} & {} F_{fca}(X,\theta )= \sigma (W_{2}\delta (W_{1}[DCT(Group(X))])) \end{aligned}$$

10 $$\begin{aligned}{} & {} Y=F_{fca}(X,\theta )X \end{aligned}$$

### Proposed model

**Figure 4:**
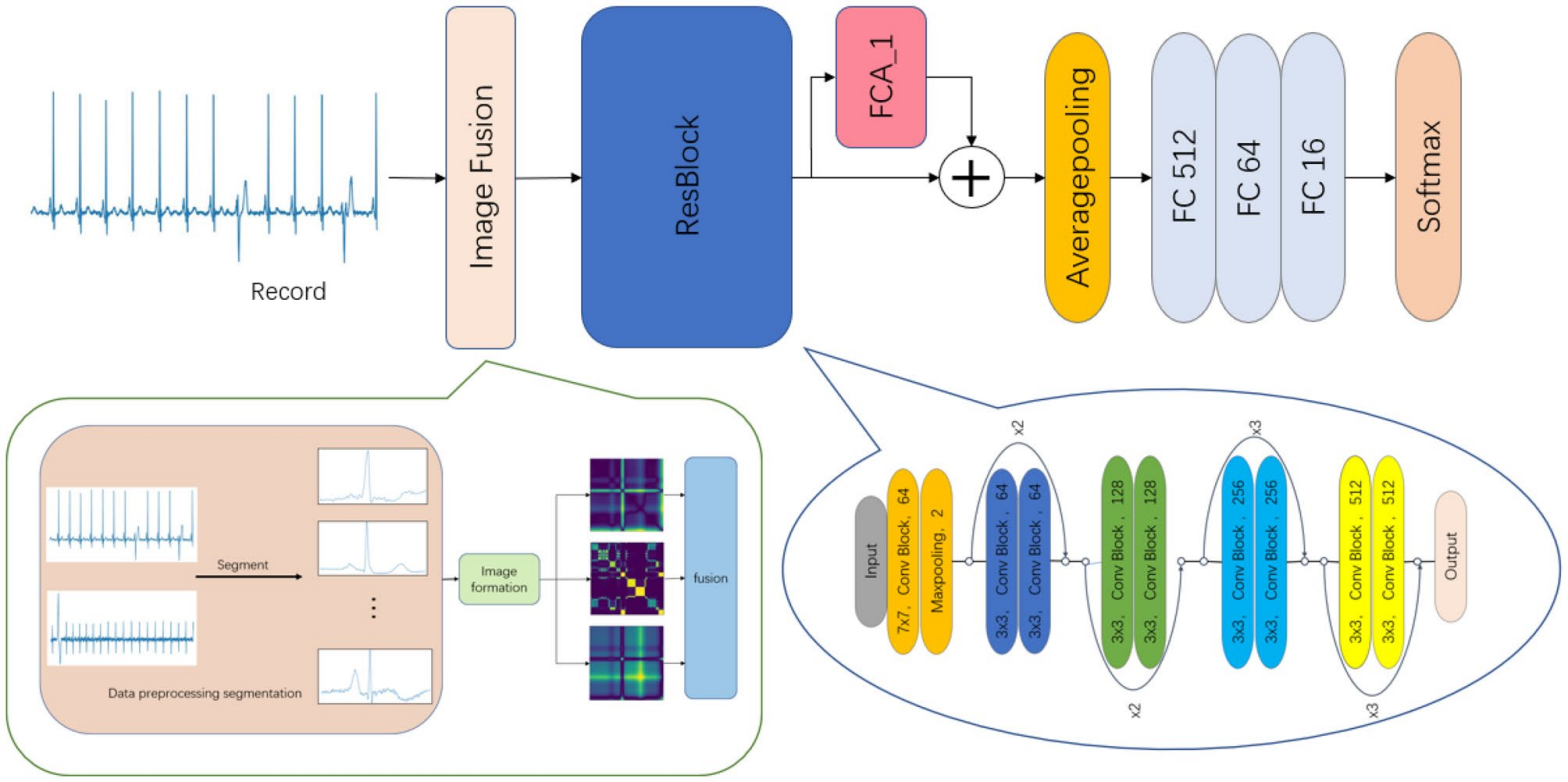
The proposed model.

**Figure 5:**
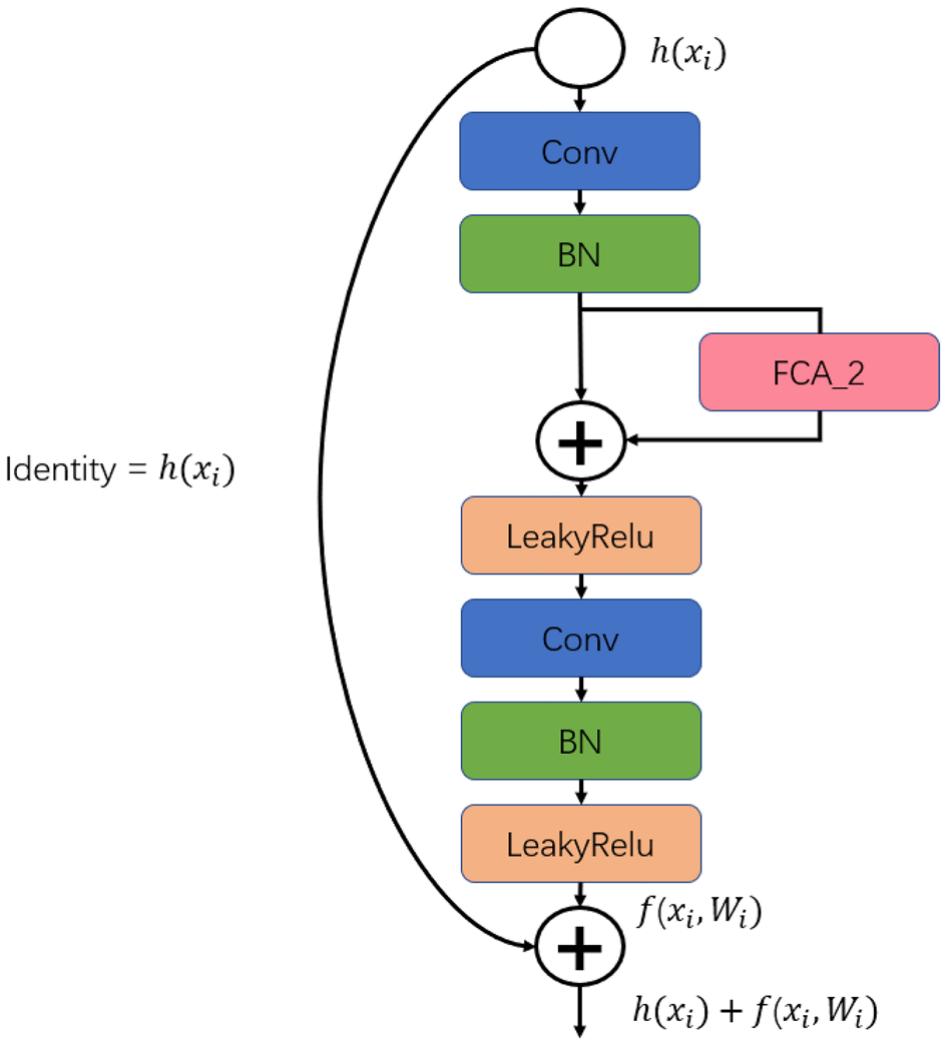
The structure of Conv block.

The proposed network architecture, depicted in Fig. 4, comprises an image fusion module, residual blocks, FCA modules, an average pooling layer, and a fully connected layer. The initial ECG data undergo segmentation into individual heartbeats and denoising through bandpass filtering to generate the raw inputs for the network. Within the network, the image fusion module converts the initial data into three image variants (RP, GAF, MTF) and merges them along the channel axis for acquiring comprehensive high-dimensional feature data via channel-wise concatenation. Subsequently, the processed data flows into ResBlocks post image fusion, comprising a convolutional layer, a pooling layer, and four convolutional blocks. The arrangement for each convolutional block is delineated in Fig. 5.

The primary objective of the initial convolutional and pooling layers is to extract fundamental features. Building upon the shortcut concept of ResNet, the Conv blocks define $Identity=h(x_{i})$, serving to not only deepen feature extraction but also avert gradient vanishing issues. Within each Conv block, the input data undergo convolution followed by batch normalization (BN). Feature weights are then determined utilizing the FCA mechanism, with the LeakyReLU activation function applied twice within the Conv block. The processed data proceed to the subsequent convolutional layer for further treatment involving batch normalization and activation, yielding $f(x_{i},W_{i})$. Following traversal through four distinct Conv blocks, the output is channeled into an average pooling layer before engaging in fully connected operations. To curtail overfitting, a dropout layer with a 0.5 probability accompanies the FC512 and FC64 layers. Ultimately, the fully connected layers’ output undergoes classification via a softmax function. During model training, a batch size of 128 is set, alongside the usage of label smoothing loss function and Adam optimizer. The training iterations are capped at 80, with the most accurate model saved for evaluation on the test set post-training.

## Experiments

### Experimental setup

Following the guidelines outlined in the ANSI/AAMI EC57:1998 standard, four recordings of patients with pacemakers were excluded from this study. A total of 100,630 heartbeats were collected from 44 recordings in the MIT-BIH-AR database.

**Table 2:** The ECG heartbeat division strategy in this paper.

	N	S	V	F	Total number
Training-set	71,980	2224	5639	647	80,490
Test-set	18,072	544	1358	166	20,140

The dataset was partitioned into two segments: 80,490 heartbeats were allocated for training the classification model, while 20,140 heartbeats were reserved for assessing the performance of the proposed approach, as detailed in Table 2. Borderline-SMOTE was employed to oversample the training set, resulting in a final training dataset comprising 324,285 heartbeats. A ten percent subset of the original training data was randomly chosen as the validation set for hyperparameter tuning. The model training was conducted on a workstation featuring an Intel 12700 CPU, an NVIDIA GTX 3060Ti GPU, and 16 GB of memory.

### Evaluation metrics

To assess the performance of the proposed model, this study employed four statistical performance metrics: accuracy (*Acc*), sensitivity (*Se*), positive predictivity (*PPV*), specificity (*Sp*) and *F*1 score. The equations defining these metrics are presented in Eqs. (11)–(14). Within a category, TP signifies the correctly identified beats, TN denotes the accurately unidentified beats, FP encompasses misclassified beats from different categories, and FN includes beats from a particular category falsely classified into other categories^33^.

11 $$\begin{aligned} Acc(\%)&=\frac{TP+TN}{TP+FP+TN+FN}*100\% \end{aligned}$$

12 $$\begin{aligned} PPV(\%)&=\frac{TP}{TP+FP}*100\% \end{aligned}$$

13 $$\begin{aligned} Se(\%)&=\frac{TP}{TP+FN}*100\% \end{aligned}$$

14 $$\begin{aligned} F1&=\frac{2*SE*PPV}{SE+PPV}*100\% \end{aligned}$$

## Result and discussion

**Figure 6:**
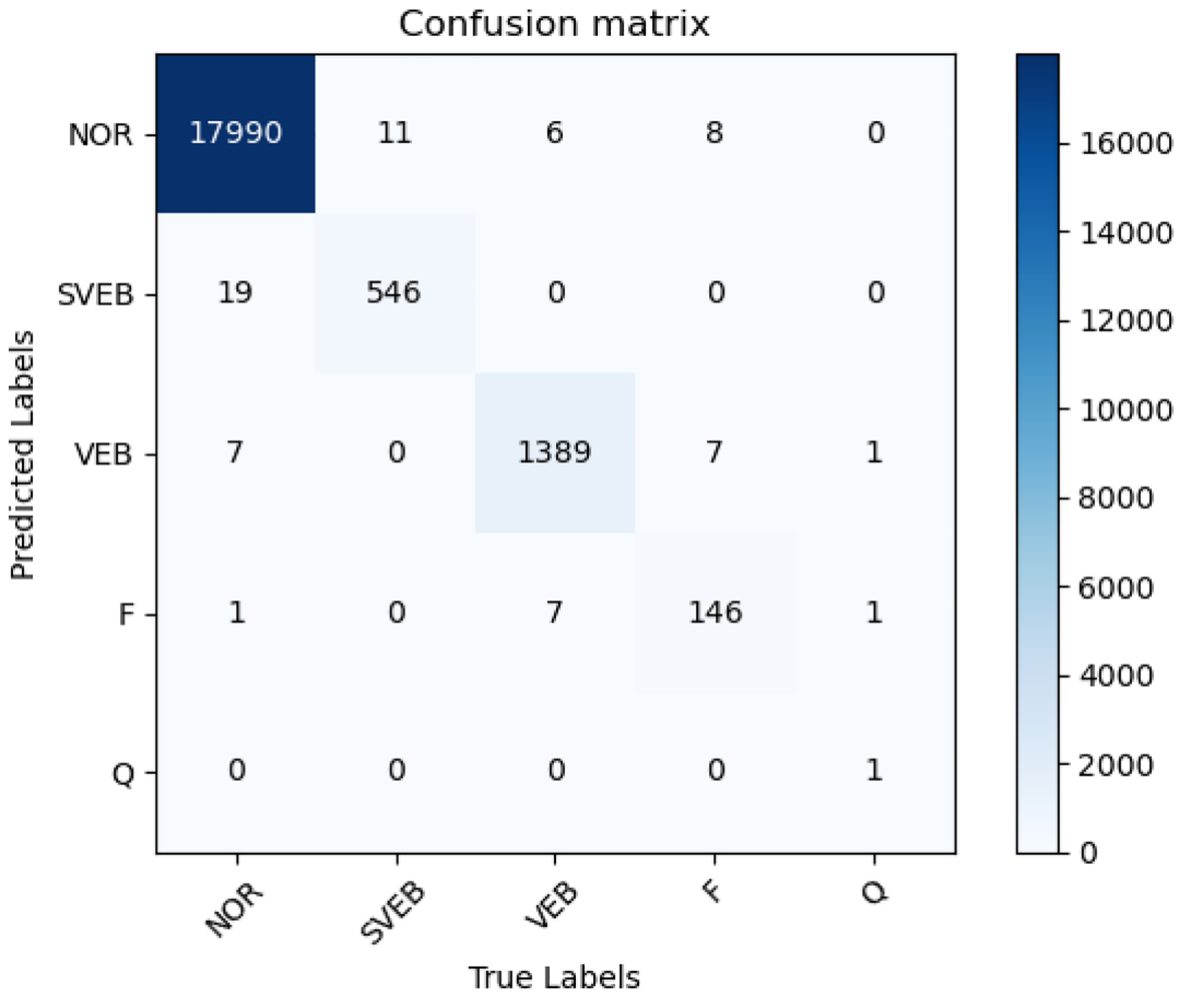
Confusion matrix yielded by the proposed method.

The performance of the proposed model on the MIT-BIH-AR database is shown in Fig. 6. The overall accuracy of the proposed model reaches 99.6% when performing 5-classification beat recognition, and it is easy to see that the misclassified beat types are mainly concentrated in classes N, S, and F. The misclassification between class N and class S may be because the feature vectors extracted by the neural network after converting 1D ECG signals into spectrograms are similar to a certain extent, which makes it easy to confuse the two types of beats that are not similar to each other, resulting in misclassification between the two types of beats. As mentioned earlier, there is a serious data imbalance in the MIT-BIH-AR database, and although Borderline-SMOTE was used to oversample the data in this study to reduce the imbalance between classes, there is still a large gap between the number of samples of different classes, and it is reasonable to assume that the main reason for the poor detection of class F is that the sample size of class F is too small. On the other hand, it is possible that the FCA is not sensitive to the feature information of class F when focusing on the channel dimensional features. In addition, due to the small sample size, although the number of misclassifications seems to be small, the proportion of misclassifications is significant. In conclusion, the proposed method has the potential for improvement in terms of feature extraction and sensitivity to feature information. The small number of Q-class heartbeats was the main reason for the misclassification effect, and Q-class heartbeats are generally unable to be identified and classified by doctors in practical applications, so they are not discussed in this paper.

### Ablation study

The proposed model integrates a residual structure with an FCA module to enhance model depth for improved identification of VEB-class and SVEB-class heartbeats. It incorporates the Borderline-SMOTE technique to address data imbalance by oversampling the data and endeavors to fuse the three image types across various dimensions. To assess the efficacy of these techniques within the model, this section conducted a single-variable control experiment from four viewpoints: model depth, hyperparameters, loss functions, and transformation methods. To illustrate the compatibility between FCA and the proposed model, we juxtapose the experimental outcomes with those of alternative attention mechanisms such as CoordAttention (CA), Squeeze-and-Excitation (SE), and Convolutional Block Attention Module (CBAM), as presented in Table 3.

The FCA module, as demonstrated in the table, effectively identified heartbeats by adaptively adjusting the weights of different frequency channels. This adaptive feature led to enhanced accuracy in heartbeat identification. Deeper model architectures outperformed shallow networks in classifying heartbeats. The conversion of heartbeats into three-channel images proved more effective than using single-channel images alone. Combining three images in the channel dimension resulted in superior accuracy compared to using a single image or conducting fusion in the height dimension. Moreover, employing the Borderline-SMOTE technique for data oversampling positively impacted the model’s heartbeat identification prowess, as illustrated in Table 3. The experimental outcomes validated the efficacy of oversampling data with the Borderline-SMOTE technique in enhancing the model’s heartbeat identification capabilities.

**Table 3:** The ablation study.

Modalities	Overall	N (%)			SVEB (%)			VEB (%)			F (%)		
	*Acc*	*Se*	*PPV*	*F*1	*Se*	*PPV*	*F*1	*Se*	*PPV*	*F*1	*Se*	*PPV*	*F*1
CA	99.3	99.5	99.8	99.6	96.4	91.6	93.9	98.6	98.4	98.5	89.4	80.9	84.9
SE	99.4	99.6	99.8	99.7	96.2	93.4	94.8	98.7	97.3	98.0	87.6	86.0	86.8
CBAM	99.3	99.6	99.8	99.7	96.6	93.1	94.8	98.6	98.0	98.3	88.8	82.2	85.4
Without Fca_1	99.1	99.7	99.5	99.6	89.4	95.8	92.5	97.8	96.8	97.3	79.5	82.6	81.0
Without Fca_2	99.2	99.8	99.5	99.6	89.9	96.0	92.8	98.1	97.2	97.6	73.3	92.9	81.9
Change number of ResBlock[1,1,1,1]	99.2	99.7	99.6	99.6	90.1	96.4	93.1	98.6	96.6	97.6	77.0	89.9	83.0
Focal_Loss	99.1	99.7	99.6	99.6	89.6	**96**.**9**	93.1	98.4	96.2	97.3	79.5	82.6	81.0
RP only	99.2	99.8	99.5	99.6	89.6	96.5	92.9	98.5	97.4	97.9	78.9	90.7	84.4
GAF only	99.1	99.7	99.5	99.6	89.9	95.1	92.4	97.8	96.5	97.1	80.7	89.0	84.6
MTF only	98.8	99.7	99.2	99.4	85.8	96.0	90.6	95.2	95.9	95.5	76.4	84.2	80.1
Proposed	**99**.**6**	**99**.**9**	**99**.**9**	**99**.**9**	**98**.**0**	96.6	**97**.**3**	**99**.**1**	**98**.**9**	**99**.**0**	**90**.**7**	**94**.**2**	**92**.**4**

Significant values are in bold.

### Comparisons with classic machine learning models

The proposed model was compared with several other methods proposed in^34–36^, etc. As shown in Table 4. The proposed model outperformed previous methods in terms of *Acc*, *PPV* and *Se*. Overall, the proposed method surpassed the existing VEB heartbeat recognition approach, achieving higher scores in *F*1, *SPPV*, and *Se* compared to other methods. The *F*1 score was 0.8% higher than the top method proposed by Liu et al.. Additionally, its *PPV* was higher than that of the currentbest method by 1%, and the *Se* score is also the highest. The insufficient recognition of class F, as discussed earlier in this section, can be attributed to a limited number of samples or high similarity among samples, hindering the model from comprehensively learning the characteristic information of this class during training. Furthermore, the proposed model may lack sensitivity to the feature information derived from converting class F heartbeats into spectrograms, leading to inadequate recognition of class F. In terms of SVEB heartbeat recognition, the *Se* has reached 98.0%, the *F*1 score was much higher than those of other centralized methods and 7.3% higher than the current best result. Therefore, when compared to other methods in a 5-class heartbeat classification task, the proposed approach not only achieved better classification performance for individual heartbeats but also yielded smaller gaps and exhibited better overall effectiveness.

**Table 4:** The performance of our propose method compared with the previous methods.

Method	Overall	N (%)			SVEB (%)			VEB (%)			F (%)		
	*Acc*	*Se*	*PPV*	*F*1	*Se*	*PPV*	*F*1	*Se*	*PPV*	*F*1	*Se*	*PPV*	*F*1
Liu et al.^34^	99.2	97.3	95.1	96.2	90.5	91.1	90.8	98.5	97.9	98.2	**98**.**3**	**100**	**99**.**1**
Oliveira et al.^35^	95.3	97.1	97.8	97.4	76.1	56.6	64.9	93.0	95.0	–	–	–	–
Chen et al.^36^	93.1	98.4	95.4	96.9	29.5	38.4	33.4	70.8	85.1	77.3	–	–	–
Kung et al.^37^	98.6	–	-	–	75.4	88.7	81.5	96.7	97.4	97.0	–	–	–
Ince et al.^38^	98.3	–	–	–	63.5	53.7	58.1	84.6	87.4	86.0	–	–	–
Shi et al.^39^	94.2	95.3	98.9	97.1	90.7	47.9	62.7	92.9	84.5	88.5	–	–	–
Xie et al.^40^	96.5	94.3	87.7	90.0	79.7	90.6	84.8	97.1	96.2	96.6	90.8	74.1	81.6
Zhai et al.^41^	96.1	87.9	92.0	89.9	76.8	74.0	75.4	93.8	92.4	93.1	62.4	79.6	70.0
Proposed	**99**.**6**	**99**.**9**	**99**.**9**	**99**.**9**	**98**.**0**	**96**.**6**	**97**.**3**	99.1	**98**.**9**	**99**.**0**	90.7	94.2	92.4

Significant values are in bold.

## Conclusion

This study introduces an architecture that integrates the FCA mechanism, a residual block, and multimodal image fusion. 1D ECG data were converted into three distinct images using RP, GAF, and MTF methods. Subsequently, these images were fused to capture both temporal and spatial information. The images served as inputs to an enhanced residual structure incorporating the FCA mechanism and shortcut connections to assign weights to feature information, thereby enhancing the model’s performance. The data then underwent processing through four residual and average pooling modules before entering a fully connected layer and being subjected to a softmax function for classification. The proposed model attained an accuracy rate of 99.6% on the MIT-BIH-AR database. Results from the ablation study (detailed in Table 3) highlighted that the combination of the FCA mechanism with the residual block led to heightened recognition accuracy for diverse heartbeats and improved classification performance. Consequently, the proposed approach proves to be dependable and efficient for ECG classification tasks. Nevertheless, the study is not without limitations.

The transformation of raw ECG data into images through three distinct methods and their subsequent fusion lead to increased computational costs and complexities. Calculations show that using ECG as 1D data input results in a model with 9,276,493 parameters, 1.63 GFlops of Floating Point Operations (FLOPs), and 78.1M of MemR+W (MemRead + MemWrite). However, after converting the input into images created through the fusion of RP, GAF, and MTF methods, the total parameter count rises to 11,642,701, reflecting a significant increase of 2,366,208 parameters compared to the 1D data. Moreover, MemR+W increases to 4.88 GB, and FLOPs reach 104.18 GFlops, marking a more than 60-fold increase compared to working with 1D data.

The data presented above strongly supports the initial assertion in this paragraph, highlighting that while transforming 1D signals into images can enhance model classification accuracy, it also introduces considerable computational complexity that cannot be disregarded. Therefore, our upcoming research will focus on reducing the computational demands and complexities associated with converting 1D electrocardiographic data into images, as well as optimizing the model structure to minimize the number of parameters. Given the pervasive use of smartphones and smart wearable devices in contemporary society, there is a growing trend towards adopting lightweight methods that can be seamlessly integrated into wearable technology. Moreover, the importance of interpretability cannot be overstated. Tools and techniques prioritizing interpretability play a crucial role in facilitating users’ comprehension of model operations and decision-making processes. Nonetheless, while simpler model structures are favored for their interpretability, they may compromise performance quality. Striking a balance between interpretability and performance optimization stands as a key focus for future research.

## Data Availability

The datasets analysed during the current study are available in the MIT-BIH-AR database, https://www.physionet.org/content/mitdb/1.0.0/.
